# Impact of vitamin D on pathological complete response and survival following neoadjuvant chemotherapy for breast cancer: a retrospective study

**DOI:** 10.1186/s12885-018-4686-x

**Published:** 2018-07-30

**Authors:** Marie Viala, Akiko Chiba, Simon Thezenas, Laure Delmond, Pierre-Jean Lamy, Sarah L. Mott, Mary C. Schroeder, Alexandra Thomas, William Jacot

**Affiliations:** 1 Department of Medical Oncology, Institut Régional Du Cancer de Montpellier ICM, 208 Avenue des Apothicaires, Cedex-5 34298 Montpellier, France; 2 Division of Surgical Oncology, Department of Surgery, Wake Forest University School of Medicine, Winston-Salem, USA; 3 Biometry unit, Institut Régional Du Cancer de Montpellier ICM, Montpellier, France; 4 Department of Surgical Oncology, Institut Régional Du Cancer de Montpellier ICM, Montpellier, France; 5 Imagenome-labosud, Clinique BeauSoleil, Montpellier, France; 6 Holden Comprehensive Cancer Center, University of Iowa, Iowa City, USA; 7 College of Pharmacy, University of Iowa, Iowa City, USA; 8 Department of Internal Medicine Wake Forest University School of Medicine, Winston-Salem, USA

**Keywords:** Vitamin D, Neo-adjuvant breast cancer, pCR

## Abstract

**Background:**

There has been interest in the potential benefit of vitamin D (VD) to improve breast cancer outcomes. Pre-clinical studies suggest VD enhances chemotherapy-induced cell death. Vitamin D deficiency was associated with not attaining a pathologic complete response (pCR) following neoadjuvant chemotherapy (NAC) for operable breast cancer. We report the impact of VD on pCR and survival in an expanded cohort.

**Methods:**

Patients from Iowa and Montpellier registries who had serum VD level measured before or during NAC were included. Vitamin D deficiency was defined as < 20 ng/mL. Pathological complete response was defined as no residual invasive disease in the breast and lymph nodes. Survival was defined from the date of diagnosis to the date of relapse (PFS) or date of death (OS).

**Results:**

The study included 327 women. Vitamin D deficiency was associated with the odds of not attaining pCR (*p* = 0.04). Fifty-four patients relapsed and 52 patients died. In multivariate analysis, stage III disease, triple-negative (TN) subtype and the inability to achieve pCR were independently associated with inferior survival. Vitamin D deficiency was not significantly associated with survival in the overall sample; however a trend was seen in the TN (5-years PFS 60.4% vs. 72.3%, *p* = 0.3), and in the hormone receptor positive /human epidermal growth factor receptor 2 negative (HER2-) subgroups (5-years PFS 89% vs 78%, *p = 0.056*).

**Conclusion:**

Vitamin D deficiency is associated with the inability to reach pCR in breast cancer patients undergoing NAC.

**Electronic supplementary material:**

The online version of this article (10.1186/s12885-018-4686-x) contains supplementary material, which is available to authorized users.

## Background

Neoadjuvant chemotherapy (NAC) has become a standard of care in locally advanced breast cancer, especially for patients with large tumor size, lymph node metastasis, HER2 overexpression, triple negative breast cancer (TNBC) subtype, or inflammatory breast cancer. The aims of NAC are to reduce the size of the tumor to increase the breast conservation rate and to initiate an early systemic therapy especially in locally advanced breast cancer (LABC) to treat micrometastatic disease. This therapeutic approach allows an in vivo assessment of the tumor chemotherapy (CT) sensitivity using the pathological response data [1]. Systemic treatment usually consists of sequential chemotherapy regiment with anthracycline and taxanes, with the addition of trastuzumab for patients with HER2 amplified (HER2+) tumors. A relationship between chemotherapy response and survival has been suggested in some trials and confirmed in two large meta-analyses [2, 3]. Indeed, pCR is associated with improved overall survival (OS). This association appears stronger in the HER2+/ HR- disease with a pCR rate of approximately 40% [4]. Response after NAC in those patients is a strong predictor of recurrence and survival. Triple negative breast cancer patients represent a subgroup benefitting from NAC, with pCR rate of 20 to 40% [5–8]. In this subset of patients, obtaining pCR is a biomarker of improved survival. On the contrary, not attaining pCR is associated with a poor prognosis, [7].

Vitamin D (VD) has gained in interest in recent years due to its impact on cancer.

Indeed, VD seems to play a key role in the cycle cell pathway, especially in breast cancer. Preclinical data have found that VD impacts the regulation of cancer cell proliferation by intervening on the cell cycle via kinases such as cyclines, cyclin-dependant kinases and CDK physiological modulators [9]. In addition VD has an anti-proliferative effect and an anti-oxidative stress, anti-invasion and anti-angiogenesis activities [10]. Vitamin D might also have a synergistic effect on the anti-tumoral activity of some anti-neoplastic agents, such as anthracyclines, and taxanes [11]. This effect appears optimal when VD is administrated before or during chemotherapy [12]. Nevertheless, it has been proven that VD deficiency is extremely frequent in the global population, and even more prevalent in breast cancer patients [13].

In a previous trial, we confirmed those data, and showed that this deficit increases during NAC [14]. In addition, a VD supplementation during NAC appears safe and feasible [15]. Further, in a previous retrospective multicenter study, we demonstrated a statistically significant correlation between VD level at baseline and pCR in patients with LABC receiving NAC [16]. The objective of our present study was to confirm these results in a larger population by evaluating in an expanded cohort the impact of VD level on pCR following breast cancer NAC and to further analyze the association between VD level in this setting and survival.

## Methods

### Design and patients

We performed an observational, retrospective study including 327 patients treated with NAC in our Comprehensive Cancer Center in Montpellier between 2005 and 2010, and at the University of Iowa Holden Comprehensive Cancer Center between 2009 and 2015. One hundred and forty four patients were already included in a previous study published by *Chiba* et al. [16], we included 183 additional patients in this study. The decision for NAC was validated in multidisciplinary boards based on the local standard of care. Patients received sequential anthracycline and/or taxane-based chemotherapy, with the adjunction of HER2-directed therapies for HER2+ tumors (6 to 8 cycles). After completion of NAC, patients underwent breast surgery. Patients harboring HR+ tumors received the recommendation for adjuvant hormonal therapy after curative surgery and patients with HER2+ tumors received the recommendation for adjuvant trastuzumab per standard of care guidelines. Pathological response determination was made by institutional pathologists. Pathological complete response was defined as no residual invasive disease in breast and lymph nodes. Survival was defined as the date of diagnosis to the date of relapse (progression-free-survival [PFS]) or date of death (overall survival [OS]). This study was approved by the local institutional review boards.

### Selection criteria

Women treated with NAC with available (frozen) serum for VD level determination before or from the start of their CT were included. We excluded patients with metastatic disease at diagnosis, patients without an available VD serum, patients with a personal history of another cancer, or with bilateral breast cancer.

### Vitamin D analysis

Vitamin D deficiency was defined as < 20 ng/mL. Serum samples were collected at baseline of chemotherapy or at cycle 2. At Iowa samples of plasma were tested for 25, hydroxyl vitamin D using electrochemiluminescence immunoassay and multiplex flow immunoassay methodologies. In Montpellier, they were tested using the DiaSorin 25-Hydroxyvitamin D-^125^I RIA kit.

### Clinical staging and pathology

Clinical breast cancer staging was determined using the 7th edition of the American Joint Committee on Cancer (AJCC) at both institutions. At Iowa, institutional practices were to confirm lymph node involvement by biopsy of any radiographically or clinically suspicious axillary lymph nodes. In the French cohort, axillary ultrasound was not routinely performed. All breast cancer was diagnosed by biopsy. Immunohistochemistry (IHC) was used to determine estrogen receptor (ER), progesterone receptor (PR) status. For this analysis hormone receptor positivity (HR+) was defined as ≥10% expression of ER or PR on the tumor. HER2 testing was performed as per ASCO/CAP guidelines [17]. For equivocal HER2 results (2+) on IHC in situ hybridization was performed. Tumors which were HR- and HER2- were considered TNBC.

### Statistical considerations

Qualitative variables were expressed in percentage with contingency table and were compared using a Chi-2 (or Fisher’s exact test if applicable). Quantitative variables were expressed with the median and range, and were compared using the Kruskal Wallis test. The pCR was evaluated based on Sataloff and Chevalier classifications [18]. Overall survival was measured between the date of the diagnosis and the date of death, or the date of the last news. Progression free survival rate was estimated using a reverse Kaplan-Meier method and presented with its 95% CI. Log rank test was used to compare the difference between the groups. The median follow-up was estimated using a reverse Kaplan-Meier method. Multivariate analysis with logistic regression on pCR was performed to evaluate the correlation between the different parameters. All *p*-values were two-sided (significance level 5%). Statistical analyses were performed using the STATA 13 software (Stata Corporation, College Station, TX).

## Results

### Patients

All patients who met the inclusion criteria described in the Methods were included. A total of 327 patients were enrolled in our observational, retrospective, multicenter study. Median age was 50 years old. Forty-two percent of our cohort had a VD level below 20 ng/ml (Table 1). There was no difference on the VD levels depending on time of measurement (baseline or cycle 2, *p = 0.18*). Eighty-five percent of tumors (*n* = 221) were ductal carcinomas, 8.8% lobular carcinomas (*n* = 23), and 6.2% (*n* = 16) was from another histological subgroup. Pathological grade (using the Ellis and Elston-modified SBR) II and III were recorded in 45.9% (*n* = 147) and 54.1% (*n* = 173) respectively. At diagnosis, 9.5% of patients presented with cT1 (*n* = 31), 60.1% with cT2 (*n* = 196), 19.3% with cT3 (*n* = 63), and 10.1% with cT4 (*n* = 33). There was a clinical lymph node involvement (cN ≥ 1) in 52.9% of the patients (*n* = 171). Seventy three percent (*n* = 237) of patients were diagnosed with clinical stage I or II, and 27% (*n* = 88) were clinical stage III. In our cohort, 28.5% (*n* = 93) of tumors had HER2+ status (14.7% [*n* = 48] were HR-/HER2+ and 13.8% [*n* = 45] were HR+/HER2+), 43.9% (*n* = 143) were HR+/HER2-, and 27.6% (*n* = 90) were TNBC.

**Table 1:** Patient and Tumor Characteristics by Vitamin D level

	Vitamin D level		*p*
	< 20 ng/ml	≥ 20 ng/ml	
Population	42% (136)	58% (191)	
Median age	49.5	50	*0.1*
Histological type			*0.3*
Ductal carcinoma	83.9% (99)	85.9% (122)	
Lobular carcinoma	7.6 (9)	9.9% (14)	
Other	8.5% (10)	4.2% (6)	
NA	(18)	(49)	
Tumor subtypes			*0.02*
HER2+	20.6% (28)	34.2% (65)	
HR+/HER2**-**	47.1% (64)	41.6% (79)	
TNBC	32.4% (44)	24.2% (46)	
NA	0	1	
Tumor size			*0.7*
T1	12.5% (17)	9.4% (17)	
T2	56.6% (77)	62.6%(119)	
T3	19.1% (26)	19.5% (37)	
T4	11.8% (16)	8.9% (17)	
NA	0	1	
Nodal status			*0.4*
N0	43.7% (59)	19.5% (93)	
N1	46.7% (63)	43.6% (82)	
N2	8.9% (12)	5.3% (10)	
N3	0.7% (1)	1.6% (3)	
NA	1	3	
SBR grade			*0.8*
II	46.6% (62)	45.5% (85)	
III	53.4% (71)	54.5%(102)	
NA	3	4	
Clinical stage			*0.96*
I-II	72.8% (99)	73% (138)	
III	27.2% (37)	27% (51)	
NA	0	2	

Low VD level, as compared with VD sufficient level was associated significantly with HR+/HER2- (47.1% vs 41.6%) and TN disease status (32.4% vs 24.2%) (*p = 0.02*). Vitamin D level did not differ between the HR+/HER2+ and HR-/HER2+ subgroups. Only tumor subtype was significantly different by VD status at the 5% level (Table 1).

### Pathological complete response and vitamin D levels

Pathological complete response was obtained in 32.7% (*n* = 107) of the patients in our cohort. Using a logistic regression model, pCR and VD level were statistically and significantly associated (*p = 0.04*). Vitamin D deficiency was associated with the chance of not obtaining pCR (73.5% non pCR vs 26.5% pCR in the low VD group). Moreover, patients with a sufficient VD level achieved pCR in 37.2% of cases.

Pathological complete response was significantly associated with some tumors subtypes (*p < 0.01*): 45.3% of patients with HER2+ tumors achieved a pCR (62.5% in the HR-/HER2+ and 40% in the HR+/HER2+ subgroups, Additional file 1), 33% for TNBC tumors, and 21.7% in the HR+/HER2- subtype. In the HR+/HER2+ subgroups (*n* = 45/327), VD level was not statistically associated with pCR (*p = 0.08*) Additional file 2. Histopathologic grade III tumors represented 66% of pCR cases compared with 34% for the grade II (*p = 0.03*) (Table 2). Patients with low clinical stage (I or II) achieved pCR significantly more often than those affected by higher stage disease (36.3% vs 22.7%; *p = 0.02*).

**Table 2:** Correlation between pCR and clinical-pathological data: univariate analysis

	No pCR	pCR	Total
Age			
< 50	44.5% (98)	55.1% (59)	*p = 0.07*
≥50	55.5% (122)	44.9% (48)	
Tumor subtypes			
HER2+	20.5% (45)	45.3% (48)	*p < 0.01*
HR+/Her2-	54.5% (120)	21.7% (23)	
TNBC	25% (55)	33% (35)	
Grade SBR			
II	51.9% (111)	34% (36)	*p < 0.01*
III	48.1% (103)	66% (70)	
Clinical stage			
I-II	68.9% (151)	81.1% (86)	*p = 0.02*
III	31.1% (68)	18.9% (20)	
Vitamin D level			
< 20 ng/mL	45.5% (100)	33.6% (36)	*p = 0.04*
≥ 20 ng/mL	54.5% (120)	66.4% (71)	

In a multivariate analysis, pCR was significantly associated with age, clinical stage, VD level, and the HER2+ subtype (Table 3).

**Table 3:** Correlation between pCR and clinical-pathological data: multivariate analysis

pCR	OR	95% CI	*p*
Age			
< 50			
≥ 50	0.45	0.3–0.7	*0.001*
Clinical stage			
I-II			
III	0.34	0.2–0.6	*0.0001*
Histological grade (SBR)			
II			
III	1.19	0.7–1.9	*0.5*
Tumor subtypes			
HER2+	1.6	0.7–3.8	*0.2*
HR+/Her2-			
TNBC	1.0	0.5–2.3	*0.9*
VD level			
< 20 ng/mL			
≥ 20 ng/mL	0.43	0.2–0.8	*0.01*

### Survival

**Fig. 1:**
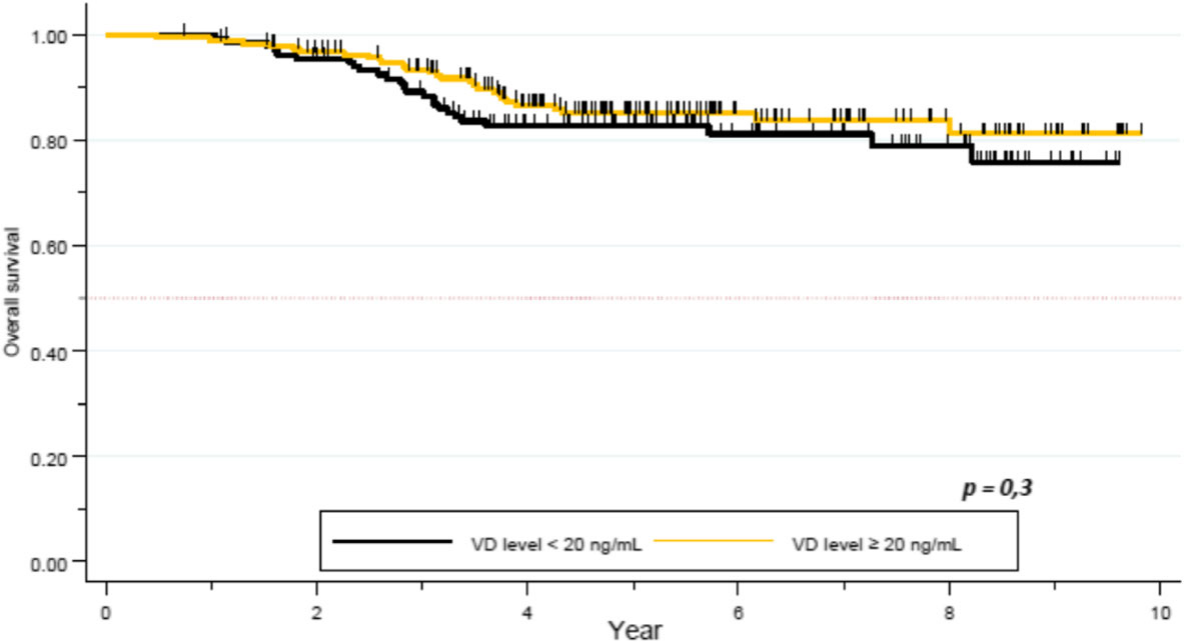
OS by Vitamin D level

After a median follow-up of 5.3 years, 54 patients relapsed and 52 patients died. Median OS was not reached. Death rate was 15.9%. One- and 5 year-OS was 100 and 83% respectively in the VD deficient group, and 99 and 85% respectively in the VD sufficient group. No difference was seen in terms of survival between these two subgroups (*p = 0.3*, Fig. 1). Five year-OS was 89% in patients with clinical stage I or II, compared to 72% for stage III. The difference was statistically significant (*p < 0.01*). There was a significant correlation between survival and pCR. Five year-OS for patients not obtaining pCR was 79% (95% CI 0.73–0.84), compared to 94% (95% CI 0.87–0.98) for those who obtained pCR (*p = 0.0007*). Ninety-one percent (95% CI 0.82–0.95) of patients with HER2+ tumors were alive at 5 years, while 92% (95% CI 0.86–0.96) for the HR+/HER2- subgroup, and 65% (95% CI 0.53–0.74) in the TNBC group. The tumor subtypes constitute an independent and significant factor for survival (*p = 0.00001,* Table 4).

**Table 4:** Correlation between OS and clinical-pathological data in a univariate analysis

	5 years-OS (%)	95%CI	*p*
Age			*0.2*
< 50	86	0.79–0.91	
≥ 50	82	0.76–0.88	
VD level			*0.3*
< 20 ng/mL	82%	0.75–0.88	
≥ 20 ng/mL	85%	0.79–0.9	
Clinical stage			*0.00001*
I-II	89%	0.84–0.93	
III	72%	0.61–0.80	
pCR			*0.0007*
no	79%	0.73–0.84	
yes	94%	0.86–0.98	
Tumor subtypes			*0.00001*
HER2+	90%	0.82–0.95	
HR+/Her2-	92%	0.86–0.96	
TNBC	65%	0.53–0.74	
SBR grade			*0.4*
II	86%	0.79–0.91	
III	83%	0.76–0.88	

In a multivariate analysis, clinical stage (*p = 0.001*), TN subgroup (*p = 0.0001*) and pCR (*p = 0.001*) were the only variables statistically correlated with OS (Table 5).

**Table 5:** Correlation between OS and clinical-pathological data in a multivariate analysis

	HR	95%CI	*p*
Age (years)			
Range (26–74)			
Median: 49.5			
< 50			
≥ 50	1.2	0.7–2.3	*0.5*
VD level			
< 20 ng/mL			
≥ 20 ng/mL	1.03	0.6–1.8	*0.9*
Clinical stage			
I-II			
III	2.8	1.6–5.0	*0.001*
Tumor subtypes			
HER2+	1.77	0.8–4.1	*0.1*
HR+/HER2-			
TNBC	6.5	3.1–13.7	*0.0001*
pCR			
no			
yes	0.2	0.09–0.5	*0.001*
SBR grade			
II			
III	0.86	0.5–1.6	*0.6*

**Fig. 2:**
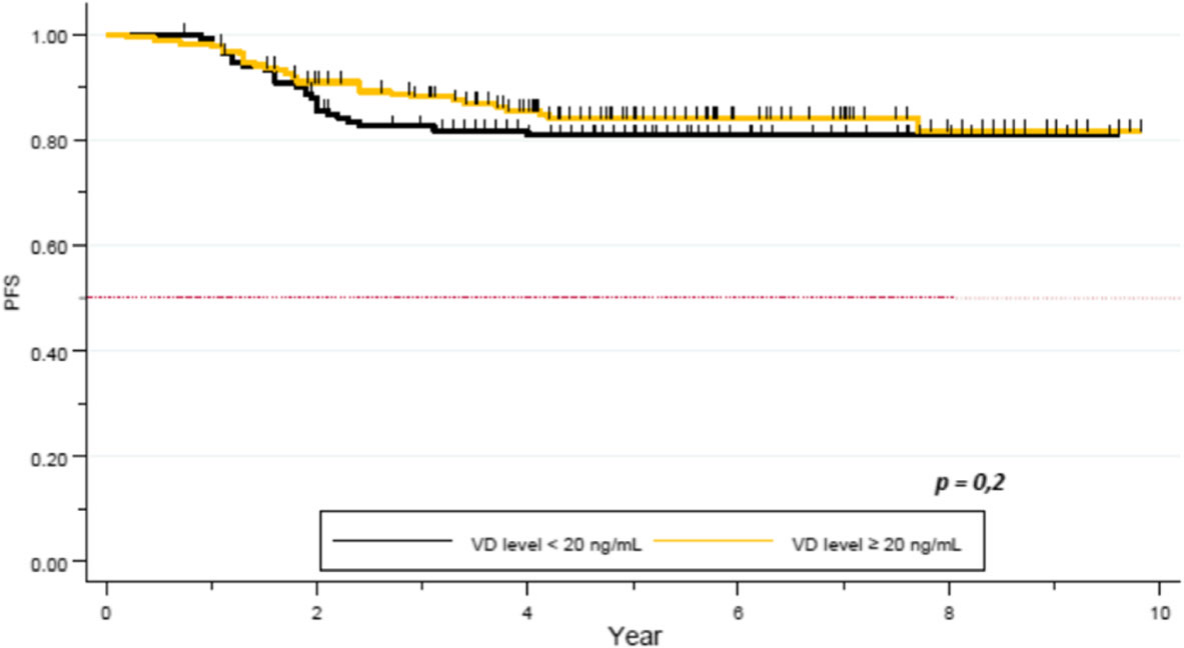
PFS by Vitamin D level in the full cohort

After a median follow up of 5.3 years, median PFS was not reached. Five year-PFS was 78% (95% CI 0.73–0.83) in our global cohort. Five year-PFS rate was 76% in the VD deficient subgroup, whereas 80% in the VD sufficient group. The difference did not achieve statistical significance (*p = 0.2*, Fig. 2). Clinical stage (84% 5-year-PFS for stages I-II and 62% for stage III) (*p = 0.00001)*, TNBC subtype (62% 5-years-PFS, *p = 0.00001*), and pathological response (72% 5- year-PFS for patients not achieving pCR, versus 92% for the pCR group, *p = 0.0002*) were significantly correlated with PFS. Other factors as histopathologic grade (*p = 0.3*), and age (*p = 0.1*) did not appear as significant factors correlated with pCR (Table 6).

**Table 6:** Correlation between PFS and clinical-pathological data in a univariate analysis

	5 years-PFS (%)	95%CI	*p*
Age			*0.1*
< 50	82	0.75–0.88	
≥ 50	75	0.67–0.81	
VD level			*0.2*
< 20 ng/mL	76	0.67–0.82	
≥ 20 ng/mL	80	0.73–0.85	
Clinical stage			*0.00001*
I-II	84	0.78–0.89	
III	62	0.51–0.72	
pCR			*0.0002*
no	72	0.65–0.78	
yes	92	0.84–0.96	
Tumor subtypes			*0.00001*
HER2+	84	0.74–0.90	
HR+/HER2-	84	0.77–0.90	
TNBC	62	0.51–0.72	
SBR grade			*0.3*
II	79	0.71–0.85	
III	78	0.71–0.84	

In a multivariate analysis, clinical stage (*p = 0.001*), TNBC subtype (*p < 0.01*) and pCR (*p < 0.01*) were the only variables significantly associated with PFS (Table 7).

**Table 7:** Correlation between PFS and clinical-pathological data in a multivariate analysis

	HR	95%CI	*p*
Age			
< 50			
≥ 50	1.4	0.84–2.3	*0.2*
VD level			
< 20 ng/mL			
≥ 20 ng/mL	0.9	0.6–1.5	*0.8*
Clinical stage			
I-II			
III	2.4	1.4–3.9	*0.001*
Tumor subtypes			
HER2+	1.6	0.30–1.21	*0.2*
HR+/HER2-			
TNBC	4.3	1.42–4.80	*0.002*
pCR			
no			
yes	0.25	0.12–0.50	*0.0001*
SBR grade			
II			
III	0.94	0.52–1.70	*0.8*

### Vitamin D and survival by tumor subtypes

**Fig. 3:**
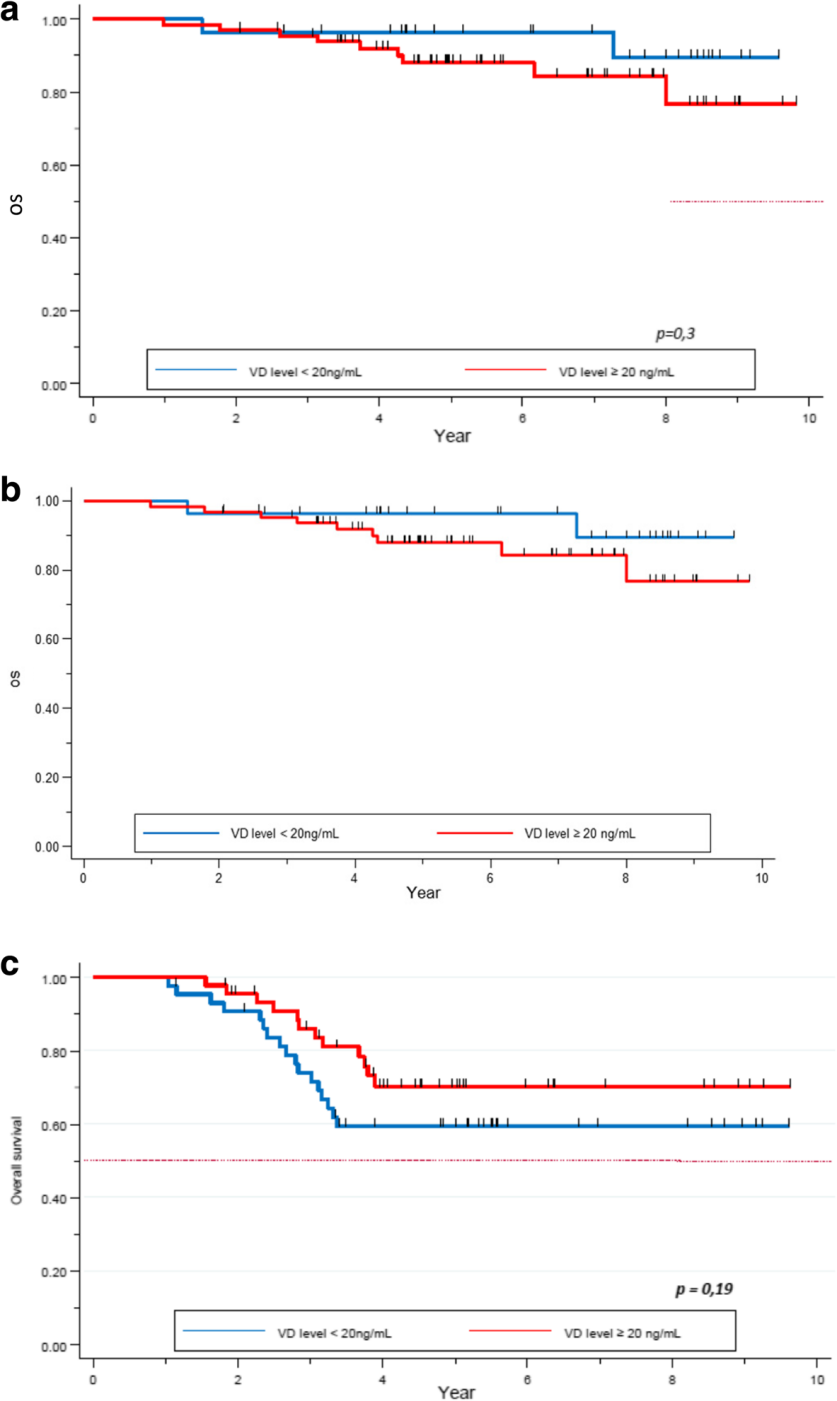
**a** OS depending on the Vitamin D level in the HER2+ tumor subtype. **b** OS depending on Vitamin D level in the HR+/HER2- tumors subtypes. **c** OS depending on the Vitamin D level in the TN tumor subtypes

Regarding OS, we found no statistical difference in the 5-year survival rate for patients with HER2+ (*p = 0.3*) and HR+/HER2- (*p = 0.8*) tumors, depending on their VD level at diagnosis (Fig. 3a, b). Regarding the TNBC subgroup, 5-year-OS was 59% (95% CI 0.4–0.7) in the VD deficient group versus 70% (95% CI 0.5–0.8) in the VD sufficient group. This trend was not statistically significant (*p = 0.2*, Fig. 3c).

**Fig. 4:**
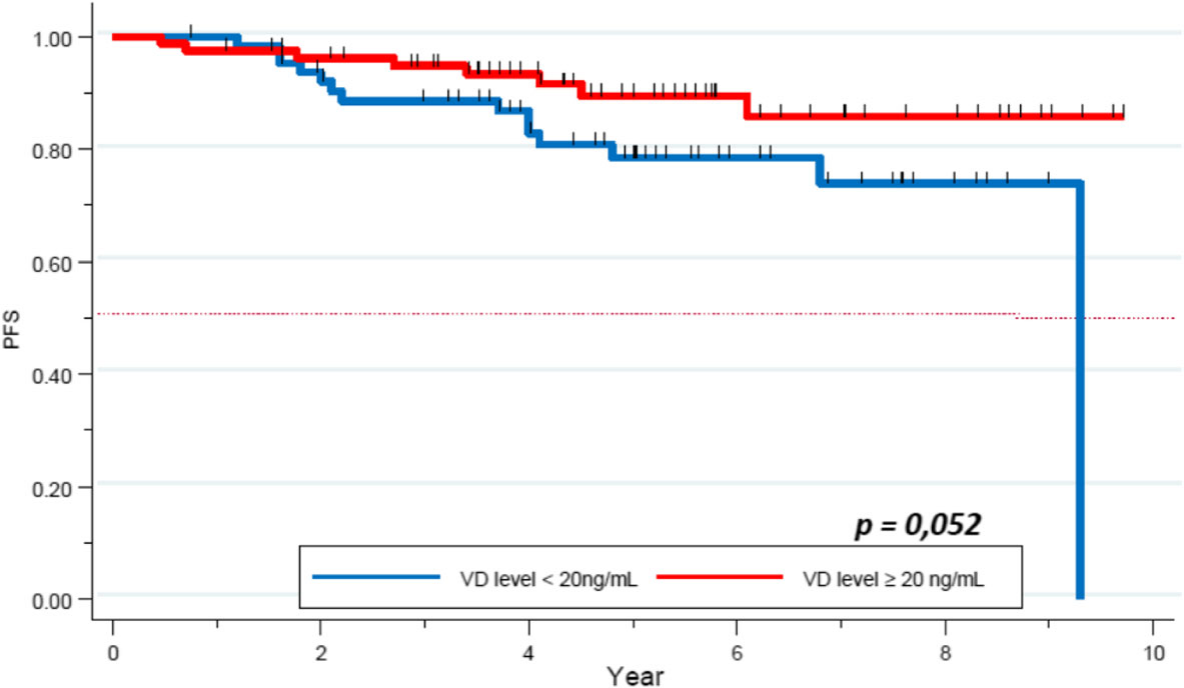
PFS depending on the VD level in the HR+/HER2- tumor subtype

**Fig. 5:**
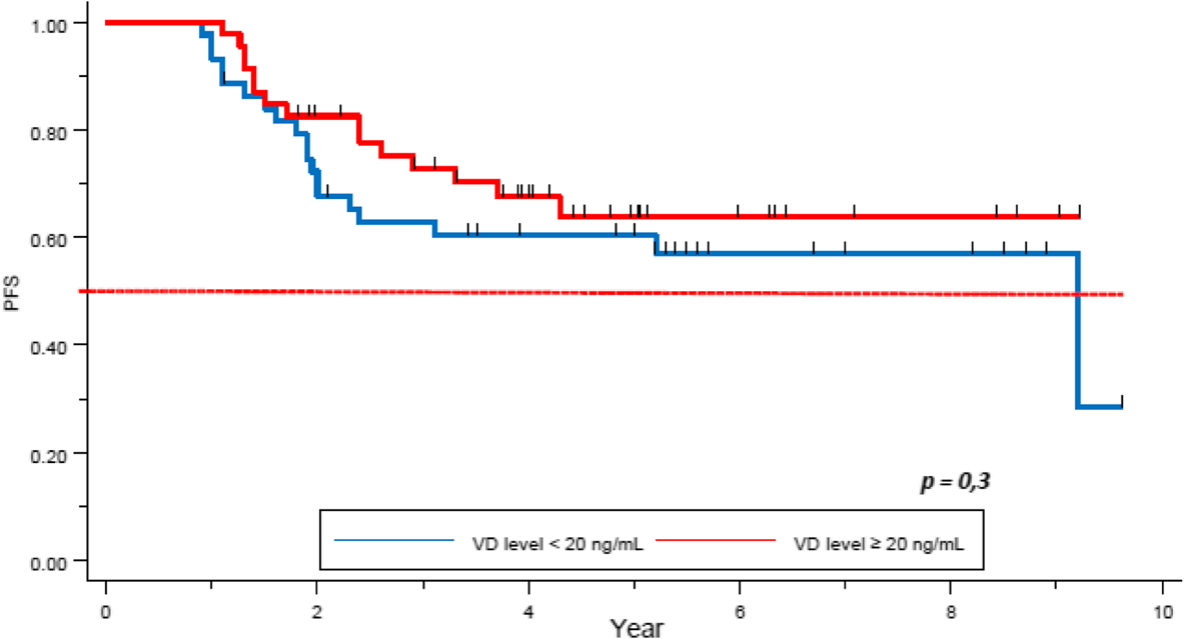
PFS depending on the Vitamin D level in the TN tumor subtypes

We analyzed PFS depending on VD level and tumor subtypes. The 5-year-PFS was of 92 and 79% in the VD deficient and the VD sufficient group respectively for patients with HER2+ tumors (*p = 0.20*). Regarding the HR+/HER2- cohort, 5-year-PFS rates were 78 and 89% respectively, this difference was approached statistical significance (*p = 0.056*), Fig. 4). Finally, a non-statistically significant trend was observed in the TNBC subgroup (60.4% vs 72.3% respectively, *p = 0.3*, Fig. 5).

### Survival and pCR depending on the profile subgroup

**Fig. 6:**
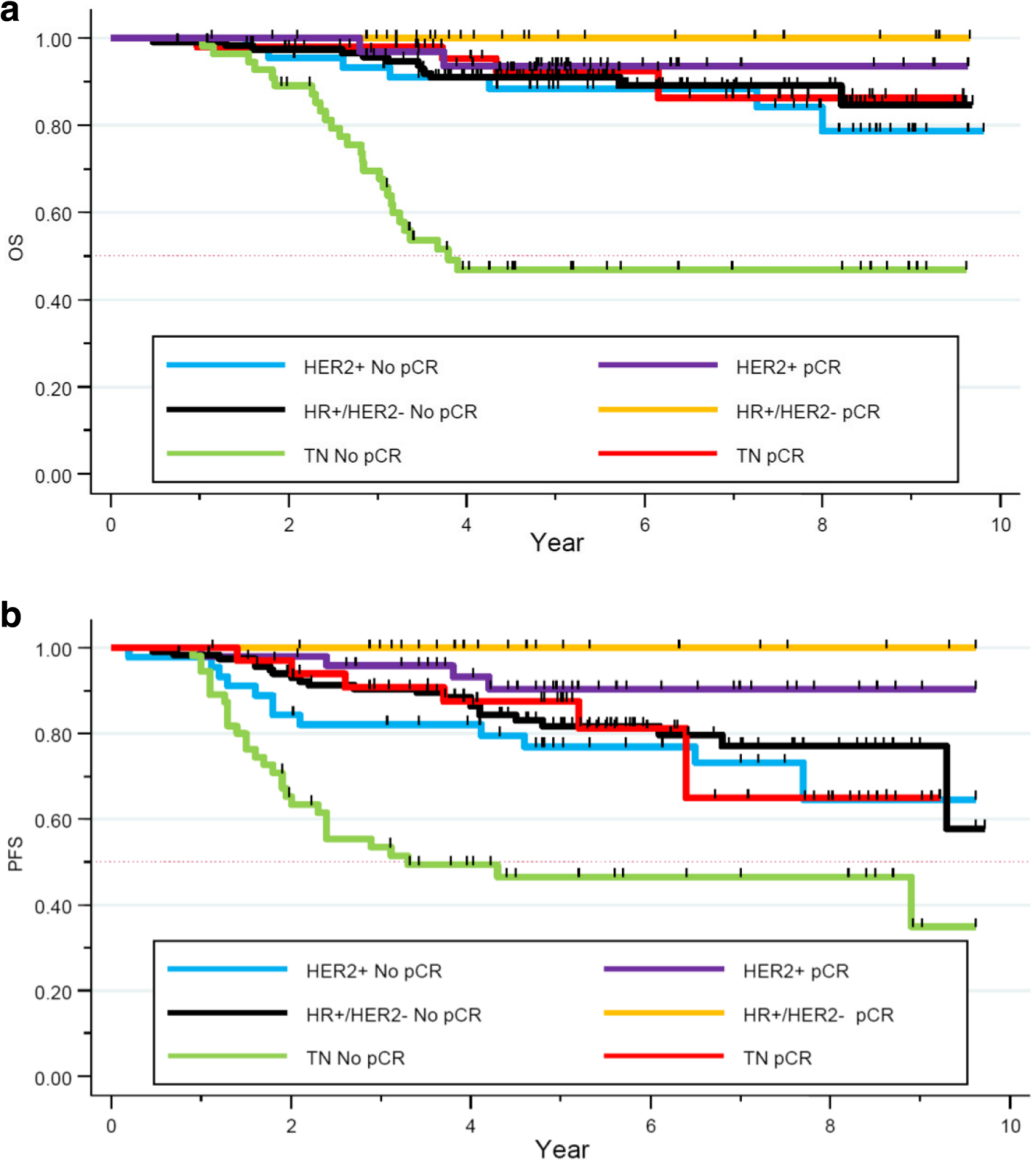
**a** OS depending on the pathological response in the different tumors subtypes. **b** PFS depending on pathological response in the different tumor subtypes

We evaluated the 5-year-OS of our cohort depending on the NAC response and their tumor subtypes. No significant difference in terms of OS was seen in the HER2+ and HR+/HER2- subgroup. Nevertheless, in the TNBC subgroup, the 5-year-OS was statistically significant (93% for patients obtaining pCR, versus 47% for non-pCR cases, *p < 0.0001*). Neoadjuvant chemotherapy response appeared as a strong and independent prognostic factor of survival in the TNBC subgroup (Fig. 6a).

Regarding PFS, 5-year-PFS rate was 77% versus 90% in the non pCR and pCR group respectively in the HER2+ subgroup (*p = 0.03*). In the HR+/HER2- cohort, 5-year-PFS rate was of 81% versus 100% in the non pCR and pCR group respectively (*p = 0.03*). Finally, in the TNBC subtype, 5-years-PFS rate for women not achieving a pCR was 46% while it was 87% for those achieving pCR (*p = 0.0009*, Fig. 6b). Pathological complete response appears as a strong and independent prognostic factor of survival, especially in the TNBC subgroup.

## Discussion

We performed a retrospective, observational, multicenter study which included 327 breast cancer patients treated by NAC. We evaluated specifically their VD level at the beginning of NAC and its impact on pCR and survival. Notably, we did not have post-NAC serial evaluations of VD levels during the 5-years follow-up.

Breast cancer patients are more frequently affected by a VD deficiency than the general population. Seventy to 80% of these patients have VD level below the lower limit of normal at breast cancer diagnosis, and that proportion even increases during NAC [13, 14, 19]. Our study confirms that patients treated by NAC frequently have deficient VD level. In fact, almost half of our cohort (42%) had baseline VD level below 20 ng/mL. Our population appears less deficient than that reported in other series (74–80% VD deficiency rate) [14, 19], however the deficiency rate is highly dependent of geographic and lifestyle variables [20]. The TNBC subtype appears to be the most affected subgroup. This result is consistent with the report published by Yao et al. [21]. Considering the VD implication in the tumorigenesis process (proliferation, apoptosis, and angiogenesis), it could be hypothesized that this deficiency might have a clinical impact on tumor response to treatment.

Few studies have evaluated the association between VD and pCR. Most of these studies did not show a significant correlation between these two factors. In the NEOZOTAC trial, a large proportion of patients were affected with low VD level at diagnosis, and even lower VD levels at the end of NAC. No correlation was seen between VD level and pCR, nevertheless, patients with sufficient VD level had a better pathological response than the others, even if this result did not achieved statistical significance [22]. Clark et al. studied, in a smaller trial, the relationship between VD and chemotherapy response. Once again, no correlation was found, but one explanation can be linked to the absence of HER2+ patients in this study [23]. Indeed, this subgroup of patients are the one responding the most frequently to chemotherapy, with the higher pCR rate, especially since the addition of trastuzumab and other HER2-directed therapies [2]. The lack of HER2+ patients in the study by Clark et al, limits interpretation of these results.

Our study confirms the significant correlation between VD level and pCR. Lower VD level significantly decreases the probability of attaining pCR. These data are consistent with our previous study [16], and validated in this expanded cohort. This results may be explained by the potential effect of VD on chemotherapeutic agents such as taxanes and anthracyclines, both of which form the backbone of breast cancer treatment [11, 24].

Tumor subtypes, histological grade and clinical stage, as expected were also associated with pCR and were found to be independent predictive factors of pCR in our population [25].

In our study, pCR was achieved in 32.4% of patients, which is higher than in the meta-analyses previously reported [2, 3, 26] (16–22% pCR rates). However, this difference may be considered altogether with the respective proportions of the biological subgroups. Additionally, our cohort is more recent than the Cortazar study, and likely benefit from improved systemic therapies, such as anti-HER2 targeted therapies and the more wide-spread use of taxanes. Consistent with previously reported literature, pCR was attained more frequently in the HER2+/HR- (60%) subtype (40% for the HR+/HER2+ one), followed by the TNBC subtype (33%) and finally the HR+/HER2- (21%) subtype.

In our cohort we observed a good prognosis, with a median PFS and OS not reached after a median 5.3 years of follow up. In the meta-analysis by Cortazar et al., pCR was suggested as a surrogate endpoint due to its correlation with survival, achieving pCR being associated with an improved survival, and a decrease risk of recurrence [2, 3]. In our study, pCR and survival are strongly associated, confirming its role as a prognostic factor, but with variable magnitude depending on tumor subtypes at this early follow-up time-point.

In the population not achieving pCR, the HR+/HER2- subgroup experienced the best prognosis, followed by HER2+ then TNBC patients. Nevertheless, for patients achieving pCR, no statistical difference was seen in the different subgroups. Pathological complete response appears as a strong prognostic factor in the TNBC subgroup. The initial general poor prognosis of this subtype is altered for patients achieving pCR (5 years-OS 93% versus 47%), as it has been initially reported by Liedtke et al. [7].

Other studies found more frequent deficiency of VD in this subgroup [21, 27]. In our study, no correlation was found between VD level and survival in this subgroup, however it appears to be a trend for a better survival in the VD sufficient group (5-year-OS of 60% in the VD deficient group versus 70% in the normal VD level one, *p = 0.2*, Fig. 3c; (5-year-PFS of 60.4% versus 72.3% in the low and normal VD level group respectively, *p = 0.3,* Fig. 4). Similar trend was seen in the study by Al-Azhri et al. [10]. This lack of statistical significance could be explained by the relatively small number of patients in our TNBC cohort. In the same article, Al-Azhri et al demonstrated that TNBC was mostly associated with a low level of VD receptor (VDR), due to a down regulation mechanism. VDR functionality is necessary for VD mediated anti-cancer activity. Indeed, in vitro, the reintroduction of VDR restored the anti-proliferative action of VD [10]. Thus, it is possible that appropriate VD levels are of greater impact in VDR functional tumors.

In addition, our analysis showed a near-significant correlation between VD level and PFS in the HR+/HER2- subgroup. It is likely that with further follow-up this finding will achieve significance at the 5% level. Some meta-analyses previously confirmed a positive association between sufficient VD level and better survival, nevertheless, no specific data was specifically available for the HR+/HER2- subgroup [28–30]. One way to explain this link could be based on the discovery of new pathways associated with VD, modulating the activity of HR+ breast cancer cells. Indeed, Krishnan et al, showed on in vitro and in vivo models that VD might decrease the expression of aromatase, and so decrease the synthesis of estrogen [31]. Thus the inhibition of estrogen synthesis and signaling by calcitriol, and its anti-inflammatory actions may play an important role in inhibiting HR+ breast cancer.

## Conclusion

In our retrospective observational study, VD level appears correlated with pCR in breast cancer patients treated with NAC. Pathological complete response is a validated, strong and independent prognostic factor of survival, especially in the TNBC population. No significant correlation was yet seen between VD level and overall survival. Nevertheless, a trend was seen in PFS in the HR+/HER- subgroup and in OS in the TNBC subgroup. Considering the natural history of the different breast cancer subgroups, the actualization of survival with a longer follow-up will allow the evaluation of the presence of similar correlations in the other breast cancer subtypes. Further studies are warranted in a larger cohort population in order to evaluate the link between VD level and survival. An interventional prospective study in this population to analyze the impact of VD supplementation on pCR and survival, eventually stratified by tumoral VDR expression would be warranted. Notably, this intervention is highly actionable and relatively inexpensive which could offer an opportunity for an easily applicable and value-based improvement in breast cancer outcomes.

## Additional files

Additional file 1: pCR rate depending on the HER2+ subtypes. (DOCX 13 kb)

Additional file 2: pCR rate depending on the VD level at baseline in the two HER2+ subgroups: a HR+/HER2+. b HR-/HER2+. (DOCX 15 kb)

## Abbreviations

AJCC: American joint committee on Cancer
CT: Chemotherapy
ER: Estrogen receptor
HER2: Human epidermal receptor 2
HR: Hormone receptor
IHC: Immunohistochemistry
LABC: Locally advanced breast cancer
NAC: Neoadjuvant chemotherapy
OS: Overall survival
pCR: Pathological complete response
PFS: Progression-free-survival
PR: Progesterone receptor
SBR: Scarff, Bloom and Richardson
TNBC: Triple negative breast cancer
VD: Vitamin D

## References

[1] Mieog JSD, van der Hage JA, van de Velde CJH. Preoperative chemotherapy for women with operable breast cancer. Cochrane Database Syst Rev. 2007:CD005002. 10.1002/14651858.CD005002.pub2. 10.1002/14651858.CD005002.pub2 PMC7388837 17443564

[2] Cortazar P, Zhang L, Untch M (2014). Pathological complete response and long-term clinical benefit in breast cancer: the CTNeoBC pooled analysis. Lancet Lond Engl.

[3] von Minckwitz G, Untch M, Blohmer J-U (2012). Definition and impact of pathologic complete response on prognosis after neoadjuvant chemotherapy in various intrinsic breast cancer subtypes. J Clin Oncol Off J Am Soc Clin Oncol.

[4] Gianni L, Pienkowski T, Im Y-H (2012). Efficacy and safety of neoadjuvant pertuzumab and trastuzumab in women with locally advanced, inflammatory, or early HER2-positive breast cancer (NeoSphere): a randomised multicentre, open-label, phase 2 trial. Lancet Oncol.

[5] Wu K, Yang Q, Liu Y (2014). Meta-analysis on the association between pathologic complete response and triple-negative breast cancer after neoadjuvant chemotherapy. World J Surg Oncol.

[6] Barton MK (2014). Bevacizumab in neoadjuvant chemotherapy increases the pathological complete response rate in patients with triple-negative breast cancer. CA Cancer J Clin.

[7] Liedtke C, Mazouni C, Hess KR (2008). Response to neoadjuvant therapy and long-term survival in patients with triple-negative breast cancer. J Clin Oncol Off J Am Soc Clin Oncol.

[8] Cortazar P, Geyer CE (2015). Pathological complete response in neoadjuvant treatment of breast cancer. Ann Surg Oncol.

[9] Verlinden L, Verstuyf A, Convents R (1998). Action of 1,25(OH)2D3 on the cell cycle genes, cyclin D1, p21 and p27 in MCF-7 cells. Mol Cell Endocrinol.

[10] Al-Azhri J, Zhang Y, Bshara W (2017). Tumor expression of vitamin D receptor and breast Cancer histopathological characteristics and prognosis. Clin Cancer Res.

[11] Hershberger PA, Yu WD, Modzelewski RA (2001). Calcitriol (1,25-dihydroxycholecalciferol) enhances paclitaxel antitumor activity in vitro and in vivo and accelerates paclitaxel-induced apoptosis. Clin Cancer Res Off J Am Assoc Cancer Res.

[12] Light BW, Yu WD, McElwain MC (1997). Potentiation of cisplatin antitumor activity using a vitamin D analogue in a murine squamous cell carcinoma model system. Cancer Res.

[13] Goodwin PJ, Ennis M, Pritchard KI (2009). Prognostic effects of 25-hydroxyvitamin D levels in early breast cancer. J Clin Oncol Off J Am Soc Clin Oncol.

[14] Jacot W, Pouderoux S, Thezenas S (2012). Increased prevalence of vitamin D insufficiency in patients with breast cancer after neoadjuvant chemotherapy. Breast Cancer Res Treat.

[15] Jacot W, Firmin N, Roca L (2016). Impact of a tailored oral vitamin D supplementation regimen on serum 25-hydroxyvitamin D levels in early breast cancer patients: a randomized phase III study. Ann Oncol Off J Eur Soc Med Oncol.

[16] Chiba A, Raman R, Thomas A (2018). Serum vitamin D levels affect pathologic complete response in patients undergoing neoadjuvant systemic therapy for operable breast Cancer. Clin Breast Cancer.

[17] Wolff AC, Hammond MEH, Hicks DG (2013). Recommendations for human epidermal growth factor receptor 2 testing in breast cancer: American Society of Clinical Oncology/College of American Pathologists clinical practice guideline update. J Clin Oncol Off J Am Soc Clin Oncol.

[18] Sataloff DM, Mason BA, Prestipino AJ (1995). Pathologic response to induction chemotherapy in locally advanced carcinoma of the breast: a determinant of outcome. J Am Coll Surg.

[19] Crew KD, Shane E, Cremers S (2009). High prevalence of vitamin D deficiency despite supplementation in premenopausal women with breast cancer undergoing adjuvant chemotherapy. J Clin Oncol Off J Am Soc Clin Oncol.

[20] Zgaga L, Theodoratou E, Farrington SM (2011). Diet, environmental factors, and lifestyle underlie the high prevalence of vitamin D deficiency in healthy adults in Scotland, and supplementation reduces the proportion that are severely deficient. J Nutr.

[21] Yao S, Sucheston LE, Millen AE (2011). Pretreatment serum concentrations of 25-hydroxyvitamin D and breast cancer prognostic characteristics: a case-control and a case-series study. PLoS One.

[22] Charehbili A, Hamdy NA, VTHBM S (2016). Vitamin D (25-0H D3) status and pathological response to neoadjuvant chemotherapy in stage II/III breast cancer: data from the NEOZOTAC trial (BOOG 10-01). Breast Edinb Scotl.

[23] Clark AS, Chen J, Kapoor S (2014). Pretreatment vitamin D level and response to neoadjuvant chemotherapy in women with breast cancer on the I-SPY trial (CALGB 150007/150015/ACRIN6657). Cancer Med.

[24] Chaudhry M, Sundaram S, Gennings C (2001). The vitamin D3 analog, ILX-23-7553, enhances the response to adriamycin and irradiation in MCF-7 breast tumor cells. Cancer Chemother Pharmacol.

[25] Alvarado-Cabrero I, Alderete-Vázquez G, Quintal-Ramírez M (2009). Incidence of pathologic complete response in women treated with preoperative chemotherapy for locally advanced breast cancer: correlation of histology, hormone receptor status, Her2/Neu, and gross pathologic findings. Ann Diagn Pathol.

[26] Berruti A, Amoroso V, Gallo F (2014). Pathologic complete response as a potential surrogate for the clinical outcome in patients with breast cancer after neoadjuvant therapy: a meta-regression of 29 randomized prospective studies. J Clin Oncol Off J Am Soc Clin Oncol.

[27] Rainville C, Khan Y, Tisman G (2009). Triple negative breast cancer patients presenting with low serum vitamin D levels: a case series. Cases J.

[28] Hu K, Callen DF, Li J, Zheng H. Circulating vitamin D and overall survival in breast Cancer patients: a dose-response meta-analysis of cohort studies. Integr Cancer Ther. 2017; 10.1177/1534735417712007. 10.1177/1534735417712007 PMC6041929 28589744

[29] Mohr SB, Gorham ED, Kim J (2014). Meta-analysis of vitamin D sufficiency for improving survival of patients with breast cancer. Anticancer Res.

[30] Vrieling A, Hein R, Abbas S (2011). Serum 25-hydroxyvitamin D and postmenopausal breast cancer survival: a prospective patient cohort study. Breast Cancer Res BCR.

[31] Krishnan AV, Swami S, Feldman D (2012). The potential therapeutic benefits of vitamin D in the treatment of estrogen receptor positive breast cancer. Steroids.

