# Supplementary material for: Impact of vitamin D on pathological complete response and survival following neoadjuvant chemotherapy for breast cancer: a retrospective study
Source: BMC Cancer. 2018 Jul 30;18:770. doi: 10.1186/s12885-018-4686-x (PMC6066931; doi:10.1186/s12885-018-4686-x)
Supplement: Supplementary file 1 — pCR rate depending on the HER2+ subtypes. (DOCX 13 kb) [file 12885_2018_4686_MOESM1_ESM.docx]

**Additional file 1:**

**pCR rate depending on the HER2+ subtypes**

|  | Tumor subtypes HER2+ | |
| --- | --- | --- |
|  | HR+/HER2+ | HR-/HER2+ |
| pCR | 40%  (n=18) | 62.5%  (n=30) |
| No pCR | 60%  (n=27) | 37.5%  (n=18) |
