# Supplementary material for: Impact of vitamin D on pathological complete response and survival following neoadjuvant chemotherapy for breast cancer: a retrospective study
Source: BMC Cancer. 2018 Jul 30;18:770. doi: 10.1186/s12885-018-4686-x (PMC6066931; doi:10.1186/s12885-018-4686-x)
Supplement: Supplementary file 2 — pCR rate depending on the VD level at baseline in the two HER2+ subgroups: a HR+/HER2+. b HR-/HER2+. (DOCX 15 kb) [file 12885_2018_4686_MOESM2_ESM.docx]

**Additional file 2: pCR rate depending on the VD level at baseline in the two HER2+ subgroups :**

1. **HR+/HER2+**

|  | VD level | |
| --- | --- | --- |
|  | < 20 | ≥ 20 |
| pCR | 18.2%  (n=2) | 47.1  (n=15) |
| No pCR | 81.8%  (n=9) | 52.9%  (n=18) |

***p= 0.09***

1. **HR-/HER2+**

|  | VD level | |
| --- | --- | --- |
|  | < 20 | ≥ 20 |
| pCR | 64.7%  (n=11) | 61.3  (n=19) |
| No pCR | 35.3  (n=6) | 38.7%  (n=12) |

***p=0.8***
